# Deep learning-based 3D OCT imaging for detection of lamina cribrosa defects in eyes with high myopia

**DOI:** 10.1038/s41598-022-26520-4

**Published:** 2022-12-23

**Authors:** Mako Ota-Itadani, Hiroyuki Takahashi, Zaixing Mao, Tae Igarashi-Yokoi, Takeshi Yoshida, Kyoko Ohno-Matsui

**Affiliations:** 1 Department of Ophthalmology and Visual Science, Tokyo Medical and Dental University (TMDU), 1-5-45 Yushima, Bunkyo-Ku, Tokyo, 113-8519 Japan; 2 R&D Division, Topcon Corporation, Tokyo, Japan

**Keywords:** Eye abnormalities, Image processing

## Abstract

The lamina cribrosa (LC) is a collagenous tissue located in the optic nerve head, and its dissection is observed in eyes with pathologic myopia as a LC defect (LCD). The diagnosis of LCD has been difficult because the LC is located deep beneath the retinal nerve fibers. The purpose of this study was to determine the prevalence and three-dimensional shape of LCDs in highly myopic eyes. Swept-source OCT scan images of a 3 × 3-mm cube centered on the optic disc were obtained from 119 eyes of 62 highly myopic patients. Each LC was manually labelled in cross-sectional OCT images along the axial, coronal, and sagittal planes. A deep convolutional neural network (DCNN) was trained with the manually labeled images, and the trained DCNN was applied to the detection of the LC in every image in each plane. Three-dimensional images of the LC were generated from the labeled image of each eye. The results showed that LCDs were detected in 12 of the 42 (29%) eyes in which an LC was visible. The LCDs ran vertically at the temporal edge of the optic disc. In conclusion, 3D OCT imaging with the application of DCNN is helpful in diagnosing LCDs.

## Introduction

The lamina cribrosa (LC) is a multilayered sieve-like structure that is composed of collagenous beams and is located within the optic nerve head. The axons of the retinal ganglion cells pass through the LC to form the optic nerve^1^. Deformations of the LC can appear as a posterior bowing, a more posterior location of the laminar insertion, a thinning of the LC, and a focal lamina cribrosa defect (LCD). The presence of LCDs has been reported to be correlated with the presence of glaucoma and high myopia^2–6^. Ophthalmoscopic examinations of the LCs are difficult because they are located under the superficial optic nerve fibers and the blood vessels on the optic disc.

Recent advances of swept-source OCT (SS-OCT) have enabled clinicians to examine deeper tissues in the ocular fundus, and LCDs can be detected in cross-sectional images. Studies of SS-OCT images show that LCDs are common in highly myopic eyes^5,6^. In addition, en face SS-OCT images can be segmented from volume cross-sectional images of LCs, and LCDs were identified as hyporeflective spots within the LC in earlier studies^6,7^. However, the entire shape of LCDs was still difficult to examine in detail because of optic disc tilts and rotations in highly myopic eyes.

At present, deep learning (DL) is being used more frequently to analyze ophthalmic images, and we have analyzed three-dimensional (3D) OCT images of the posterior vitreous cavities by combining manual labeling and DL-based auto-segmentation^8^. With this method, 3D images of LCDs were generated but they have still not been evaluated in detail.

Thus, the purpose of this study was to determine the prevalence and shapes of LCDs in highly myopic eyes. To accomplish this, we created 3D images of the LC using DL techniques, and we shall show that this application of AI training was very helpful in accomplishing our purpose.

## Methods

### Participants

The protocol of this study was approved by the Institutional Review Board (IRB) of the Tokyo Medical and Dental University. All procedures adhered to the tenets of the Declaration of Helsinki for research involving human subjects. All participants gave informed consent for this study and approved the publication of their retinal findings and images.

This was a retrospective observational case series study. The medical records of patients who were examined in the Advanced Clinical Center for Myopia at the Tokyo Medical and Dental University Hospital from January to July of 2021 were reviewed. Patients whose eyes had an axial length > 26.5 mm were included. Patients with retinal diseases except for pathologic myopia and macular atrophy due to myopic maculopathy were excluded. In addition, patients with poor quality of OCT images due to a very small or a tilted optic disc were also excluded. In the end, 119 eyes of 62 patient with high myopia were studied. All participants had undergone comprehensive ophthalmic examinations, and the clinical variables including the age and sex were collected from the medical records. The refractive errors (spherical equivalents) were measured with an autorefractor (KR-800; TOPCON, Tokyo, Japan), and the axial lengths with the IOLMaster (IOLMaster 700, Carl Zeiss Meditec AG, Jena, Germany). Color fundus photographs were taken with a fundus camera (TRC-NW8F, TOPCON, Tokyo, Japan).

### Three-dimensional OCT examinations

The optic disc was scanned with an SS OCT instrument (Toriton; Topcon, Tokyo, Japan) over a field of 3 $\times$ 3 mm^2^ with the eye at the primary eye position. We defined a LCD as a loss of high reflectivity from the anterior–posterior border of the full thickness LC in the vertical, horizontal and axial serial B-scan images^6^. Cross-sectional scans of 3 mm length centered on the optic disc were also recorded to evaluate the laminar structure that allowed distinguishing LCDs from the hyporeflective regions that were clearly not LCDs, e.g., vessel shadows and pores of the LC.

### Manual labelling and AI-based auto-segmentation for LC in SS-OCT images

**Figure 1:**
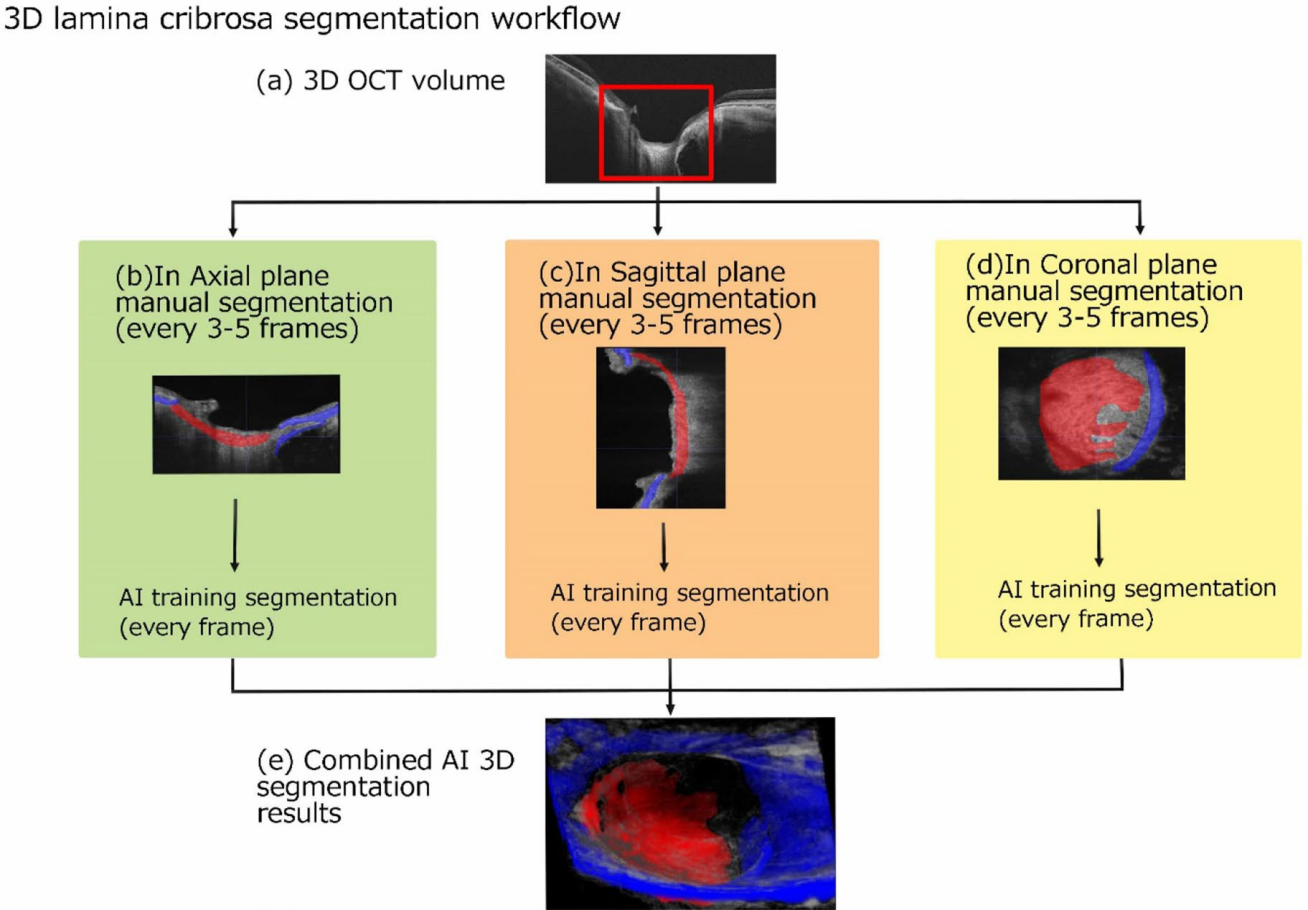
Workflow of deep learning-based 3D OCT imaging of the lamina cribrosa (LC) and LC defects (LCDs). Top row; (**a**) Representative horizontal scan of original 3D SS-OCT image is shown with the area surrounded by a red square where the segmentation was performed. Middle row; Representative images of manual segmentation in the axial (**b**), coronal (**c**), and sagittal (**d**) planes are shown. The lamina cribrosa (LC) is colored red and the peripapillary sclera (PPS) blue. Artificial intelligence (AI) training and segmentation are applied. Bottom row; (**e**) A 3D LC image was generated from the combined AI 3D segmentation results using Fiji^9^ (Version 2.1.0/1.53 k).

An AI-based segmentation procedure that was developed and published by Ohno-Matsui et al^8^ was used to segment the LCs of the 3D OCT images. To maximize the performance, an optimized deep convolutional neural network (DCNN) was trained per plane for each eye. A summary of the procedure is: first, two-dimensional cross-sectional OCT data were extracted from the 3D OCT images as 256, 222, and 190 series of images along the axial, sagittal, and coronal planes, respectively, for each patient. The entire LC was manually labelled in red and the peripapillary sclera (PPS) in blue in the cross-sectional images along the axial, coronal, and sagittal planes every 3–5 frames by a single image reader (MOI). The number of available annotated images varied from eye to eye due to the difference in the presence of the LC. Overall, 40–70 image per plane per eye were annotated. Next, a DCNN per plane was trained with the manually annotated images as the ground truth of each eye. Then, the non-labelled cross-sectional images were labelled by the trained DCNN for each plane. Finally, the 3D images of the LCs were generated from the series of cross-sectional images with continuous labels using the Fiji software^9^ (Version 2.1.0/1.53 k, Link: https://imagej.net/software/fiji/; Fig. 1). As the detailed procedures and accuracies of AI segmentation and 3D OCT imaging have been reported previously^8^, they were not reassessed in the present study.

## Results

### Demographics

Twenty-six of the 119 eyes were excluded because the lamina cribrosa was not visible due to small size or tilting of the optic disc. In addition, other 51 eyes were excluded because of poor image quality. The 3D images of the LCs were good and were analyzed in 42 eyes of 33 patients. The average age of these patients was 52.7 ± 11.6 years. The average axial length of these eyes was 30.35 ± 1.66 mm. The characteristics of the eyes with and without an LCD are shown in Table 1.

**Table 1:** Demographics of highly myopic eyes with and without lamina cribrosa defects.

	Lamina cribrosa defects	
	Present	Absent
No. of eyes (%)	12 (29%)	30 (71%)
Age (years), Mean ± SD	50.7 ± 7.79	53.5 ± 12.8
Sex (male/female)	5/7	9/21
Axial length (mm), Mean ± SD	30.8 ± 1.17	30.1 ± 1.78
Refractive error (diopters), Mean ± SD	−15.8 ± 3.38	−14.0 ± 4.77

SD = standard deviation.

LCDs were present in 12 (29%) of the 42 eyes, and the LCDs were present at the temporal region of the optic disc in 11 (92%) of the 12 eyes. In the other eye, a triangular-shaped LCD was present at the inferior-temporal region of the optic disc. The LCDs were bordered by the PPS.

### Representative cases

**Figure 2:**
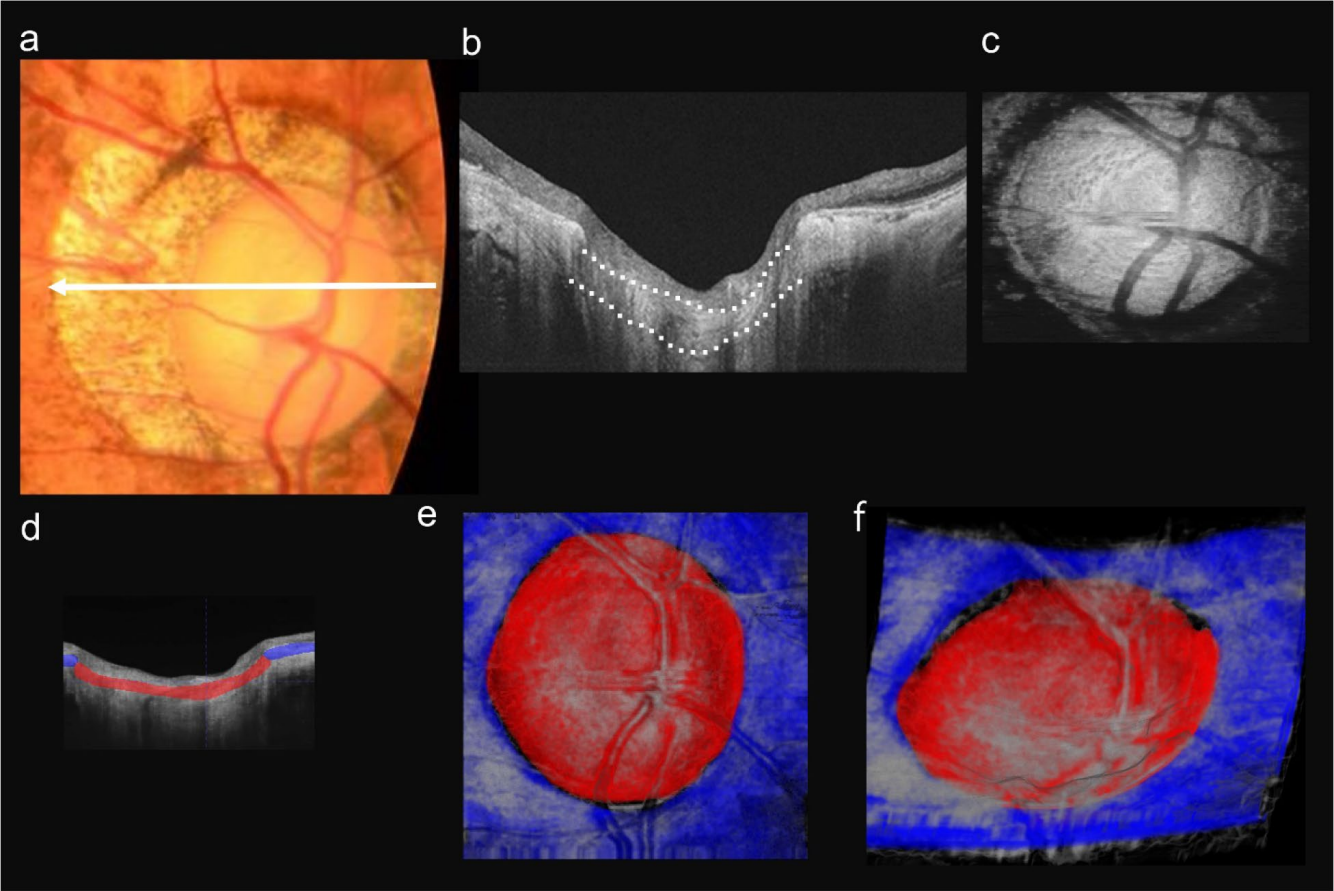
Color fundus photographs and OCT images of a 61-year-old highly myopic woman without a lamina cribrosa defect (LCD). (**a**) Color fundus photograph showing the optic disc of the right eye. A myopic conus is present in the peripapillary area temporal to the optic disc. White arrow shows scanning line for the OCT images. (**b**) Horizontal OCT cross-sectional image showing the lamina cribrosa (LC) (white dotted lines). (**c**) En face OCT image of the optic disc at the level of the LC. The LC appears as a round hyperreflective area and laminar pores are seen as hyporeflective dots within the area of the LC. The temporal edge of the LC is unclear due to signal attenuation by the surrounding tissues. (**d**) OCT image as (b) with manual labels showing the PPS colored blue and the LC colored red. (**e**,**f**) 3D images generated by the trained deep convolutional neural network. (**e**) En face 3D image shows that the LC is surrounded by the PPS. (**f**) 3D image viewed from inferior side of fundus shows that the LC is bent against the PPS curvature and forms the optic disc cup.

A representative case without an LCD is shown in Fig. 2. This 61-year-old female patient was highly myopic in her right eye. Her axial length was 29.38 mm and the refractive error was −10.37 diopters (D). The LC appeared as a round hyperreflective area in the en face OCT image, and the laminar pores were seen as hyporeflective dots within the area of LC. The temporal border of the LC was not clearly seen due to an attenuation of the signals by the surrounding tissues (Fig. 2c). The LC was colored red and the PPS blue in the 3D image of the optic disc (Fig. 2e,f). The LC was connected smoothly to the surrounding PPS and there was no dehiscence between the two tissues. In Supplementary Video 1, the generated three-dimensional (3D) image is rotated 90 degrees from an axial to a coronal point of view.

**Figure 3:**
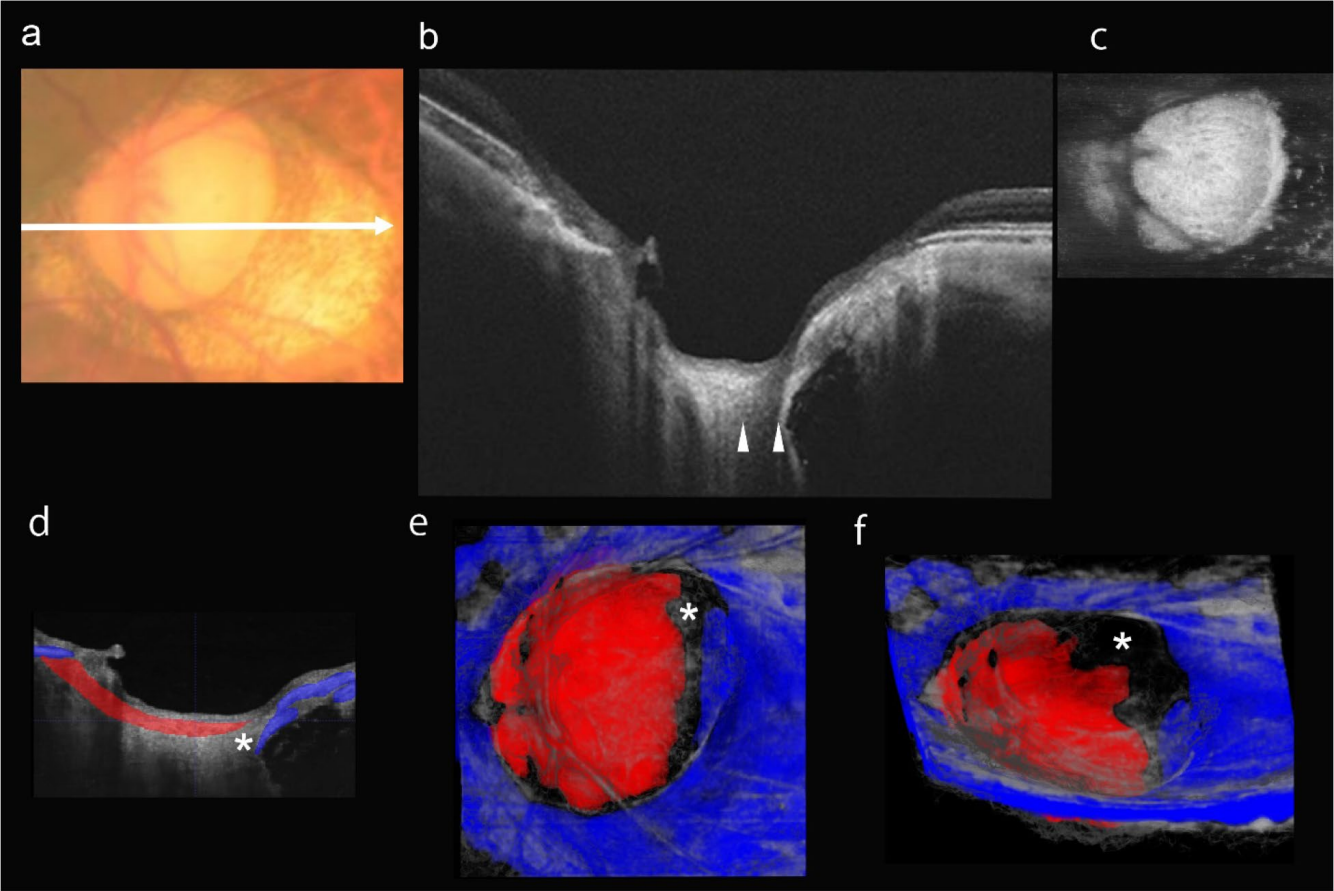
Color fundus photographs and OCT images of a 57-year-old highly myopic woman with an lamina cribrosa defect (LCD). (**a**) Color fundus photograph showing optic disc of the left eye. Myopic conus is present at the peripapillary area mostly temporal to the optic disc. White arrow shows scanning line of OCT images. (**b**) Horizontal OCT cross-sectional image showing the LCD. White arrowheads show the edges of the LCD. (**c**) En face OCT image of optic disc at the level of the lamina cribrosa (LC) showing that the temporal part of LC has lower reflectivity than the other parts of the LC. The temporal edge of the LC is blurry due to the presence of a hyporeflective area. (**d**) Height adjusted OCT image as (b) with manual labels showing the PPS (blue) and LC (red). (**e,f**) 3D images generated with the trained deep convolutional neural network. (**e**) En face 3D image with asterisk indicating the defect and showing that the LCD runs vertically at the temporal edge of the optic disc. (**f**) 3D image viewed from the inferior side of the fundus with an asterisk marking the LCD and showing that the edge of the defect is rough.

The second case was a 57-year-old woman with highly myopic left eye with an LCD (Fig. 3). The axial length of the left eye was 29.73 mm and the refractive error was −11.25 D. The color fundus photograph showed that there was no evident lesion on the temporal part of the optic disc. However, the en face OCT images showed that the temporal part of the LC had a lower reflectivity than the other parts of the LC. It was difficult to determine the exact boundary of the LC in the en face image (Fig. 3c). The LC was colored red and the PPS was colored blue in the 3D image of the optic disc. There was an unpainted area (Fig. 3d,f, asterisk) between the LC and the PPS which indicated a disinsertion of the LC from the PPS. The LCD ran vertically at the temporal part of the optic disc. In the Supplementary Video 2, the generated 3D image is rotated 90 degrees from axial to coronal point of view.

**Figure 4:**
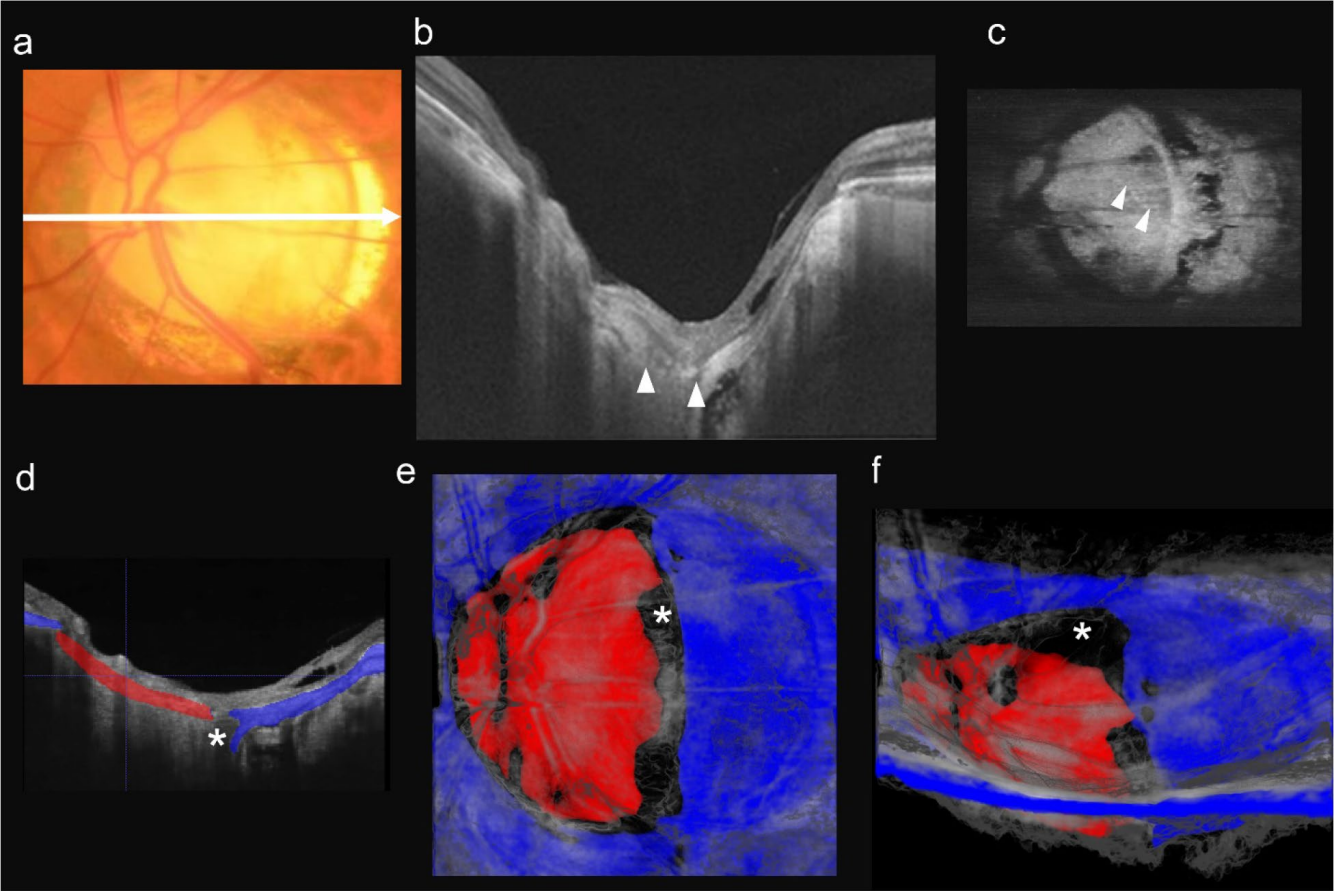
Color fundus photograph and OCT images of a 55-year-old highly myopic man with a lamina cribrosa defect (LCD). (**a**) Color fundus photograph showing the optic disc of the left eye. Myopic conus is present in the peripapillary area especially at the temporal part to the optic disc. White arrow shows scanning line for OCT images. (**b**) Horizontal OCT cross-sectional image showing the LCD. White arrowheads show the edges of the LCD. (**c**) En face OCT image of the optic disc at the level of the lamina cribrosa (LC) showing that the whole structure of the LCD is not clearly imaged due to a tilting of the optic disc. The LCD is pointed to by the white arrowheads and is seen as hyporeflective spots with unclear boundary. (**d**) Height adjusted OCT image as (b) with manual labels showing the PPS (blue) and lamina cribrosa (red). (**e,f**) 3D images generated with the trained deep convolutional neural network. (**e**) En face 3D image with asterisk indicating the defect showing that the LCD runs vertically at the temporal edge of the disc. **f:** 3D image viewed from the inferior side of the fundus with asterisk pointing to the LCD showing that the LCD appears to be torn apart from the edge of the disc.

The final case was a 55-year-old man whose left eye was highly myopic and was a representative of eyes with an LCD (Fig. 4). The axial length was 28.31 mm and the refractive error was −11.37 D. There was no lesion evident in the temporal region of the optic disc in the color fundus photograph. Because the optic disc was tilted temporally, the entire structure of the LC was not clear in the en face OCT image (Fig. 4c). The LCDs were observed as hyporeflective spots with blurred boundaries. The LC was labelled in red and the PPS in blue in the 3D image of the optic disc. It was then possible to see the uncolored areas (Fig. 4d,f, asterisk) at the temporal edge of the disc which was between the LC and the PPS. In the Supplementary Video 3, the generated 3D image is rotated 90 degrees from axial to coronal point of view.

## Discussion

The LC is located in the tissues of the optic nerve head, and it is difficult to analyze in 3D due to signal attenuation by the overlying tissues. LCDs have been detected in cross-sectional images and identified as hyporeflective spots in the highly reflective LC in en face volume images in earlier studies^6,7,10,11^. Because the size and shape of the optic disc vary in highly myopic eyes^12^, it is difficult to diagnose LCDs with only conventional cross-sectional and en face volume OCT images.

With the advent of AI-driven auto-segmentation, 3D images of the LC have been created. We are unaware of any studies which reported that each LCD can be evaluated in a single 3D image, and that the LCDs are mostly oriented vertically between the temporal region of the optic disc and the PPS. Combined with conventional en face OCT images of the optic disc, this newly developed method will provide additional information for a better understanding of the shapes and locations of LCDs.

In earlier studies, LCDs were detected in 2 to 27.8% of highly myopic eyes^5,6,13^. Our results demonstrated that 29% of eyes with high myopia have LCDs which is compatible with the results of earlier studies.

The location of the LCDs within the optic disc of highly myopic eyes has not been determined. Previously, Kimura et al. investigated whether LCDs were present in the cross-sectional images of 75 highly myopic eyes with primary open angle glaucoma. They reported that LCDs were present in the temporal region of the LC in all 75 eyes^6^. Our results showed that LCDs were located mostly at the temporal edge of the optic disc in the 3D images which is compatible with the results of Kimura et al.

The differences in the locations of the LCDs between glaucomatous eyes and highly myopic eyes have not been determined. Miki et al. reported that LCDs were in the inferior half of the optic nerve head in 63.6% of glaucomatous eyes but in contrast, LCDs were found in the inferior half of the optic nerve head in 46.4% of highly myopic eyes with glaucoma^5^. Our images of the LCs in highly myopic eyes showed that LCDs developed symmetrically in the vertical direction at the edge of the disc detached from the PPS. This was not clearly detected in the evaluation of cross-sectional images and en face volume images. These findings suggest that the clinical course of LCDs in highly myopic eyes differs from that in glaucomatous eyes.

The exact mechanism causing the development of LCDs has not been determined. Previous studies have suggested that there was a mechanical stress on the LC by the myopic morphological changes that leads to a deformation of the LC. Earlier studies reported that the presence of LCDs in highly myopic eyes was associated with myopia-related factors, such as axial length elongations, enlargement of optic disc area, ovality and tilting of optic disc, and larger area of papillary atrophy (PPA)^6,7,13^. Previous histological studies have shown that the biomechanical properties of the PPS were related to the biomechanics of the LCs^14,15^. The thickness of the LC in myopic eyes decreases as the PPA width increases^14,15^. A previous biomechanical study suggested that missing LC connective tissues can mitigate the IOP-induced neural tissue insult which suggests that the role of the LC connective tissues is more complex than simply fortifying against the IOP^16^. In this study, the eyes with LCDs had large temporal PPAs and tilted optic discs (Figs. 3,4). We suggest that the thinner LC of myopic eyes and larger PPAs makes the LC more vulnerable to horizontal mechanical stress by the changes in the optic disc morphology which contributes to the formation of LCDs. To understand the relationship between the biomechanical properties and structure of the LC in the development of LCD in highly myopic eyes, further longitudinal studies are needed.

This study has several limitations. First, the sample size was small because the aim of this study was to establish a method of constructing 3D LCD images. Second, the presence of LCDs was determined by a single grader, then the precision and recall of our methods were not evaluated. For investigations of the nasal region of the optic nerve head, manual labelling might have been relatively more challenging compared to the other parts of the disc especially in eyes with a tilted optic disc. This was because the overlying optic nerve fibers are thick in the nasal region and they and the retinal vessels overlie the LCs, which made the deeper structure poorly visible. Third, a DCNN was trained for each eye instead of trying to train one model that can be applicable for all 42 eyes because of the small number of datasets. Thus, our future goal is to create more generalized 3D OCT imaging for LCDs that can be used for all eyes in a large case series.

In conclusion, manual labelling of LCs in the 3D SS-OCT images and the application of deep convolutional neural network analyses were used to construct 3D images of the LC. This allowed us to detect LCDs and evaluate their structure. LCDs mostly ran vertically in the temporal region of the optic disc. These findings suggest that the temporal edge of optic disc was susceptible to be impaired in highly myopic eyes.

## Supplementary Information

Supplementary Video 1.

Supplementary Video 2.

Supplementary Video 3.

Supplementary Information 1.

## Data Availability

The datasets generated during and analyzed during the current study are available from the corresponding author on reasonable request.
