# Supplementary material for: Deep learning-based 3D OCT imaging for detection of lamina cribrosa defects in eyes with high myopia
Source: Sci Rep. 2022 Dec 23;12:22195. doi: 10.1038/s41598-022-26520-4 (PMC9789076; doi:10.1038/s41598-022-26520-4)
Supplement: Supplementary file 4 — Supplementary Information 1. [file 41598_2022_26520_MOESM4_ESM.docx]

The description of the supplementary videos

Supplementary video 1 shows the 3D image of the lamina cribrosa (LC) colored red and peripapillary sclera (PPS) colored blue of the 61-year-old highly myopic woman (the same patient as shown in the Figure 2) without an lamina cribrosa defect (LCD) generated by the trained deep convolutional neural network. In this video, the generated three-dimensional (3D) image is rotated 90 degrees from axial to coronal point of view.

Supplementary video 2 shows the 3D image of the LC colored red and PPS colored blue of the 57-year-old highly myopic woman with an LCD (the same patient as shown in the Figure 3) generated by the trained deep convolutional neural network. In this video, the generated 3D image is rotated 90 degrees from axial to coronal point of view. The LCD runs vertically at the temporal edge of the optic disc.

Supplementary video 3 shows the 3D image of the LC colored red and PPS colored blue of the 55-year-old highly myopic man with an LCD (the same patient as shown in the Figure 4) generated by the trained deep convolutional neural network. In this video, the generated 3D image is rotated 90 degrees from axial to coronal point of view. The LCD runs vertically at the temporal edge of the optic disc.
